# Detection and genomic characterization of hepatitis E virus genotype 3 from pigs in Ghana, Africa

**DOI:** 10.1186/s42522-020-00018-3

**Published:** 2020-07-20

**Authors:** Philip El-Duah, Dickson Dei, Tabea Binger, Augustina Sylverken, Robert Wollny, William Tasiame, Samuel Oppong, Yaw Adu-Sarkodie, Benjamin Emikpe, Raphael Folitse, Jan Felix Drexler, Richard Phillips, Christian Drosten, Victor Max Corman

**Affiliations:** 1 Charité-Universitätsmedizin Berlin, Humboldt-Universität zu Berlin, Berlin Institute of Health, Institute of Virology, Berlin, Germany; 2 Kumasi Centre for Collaborative Research in Tropical Medicine, Kumasi, Ghana; 3 Ghana Veterinary Service, Kumasi, Ghana; 4 School of Veterinary Medicine, Kwame Nkrumah University of Science and Technology, Kumasi, Ghana; 5 Department of Theoretical and Applied Biology, Kwame Nkrumah University of Science and Technology, Kumasi, Ghana; 6 Institute of Virology, University of Bonn Medical Centre, Bonn, Germany; 7 Department of Wildlife and Range Management, Kwame Nkrumah University of Science and Technology, Kumasi, Ghana; 8 Department of Clinical Microbiology, Kwame Nkrumah University of Science and Technology, Kumasi, Ghana; 9 German Centre for Infection Research, Berlin, Germany

**Keywords:** Foodborne diseases, One health, Zoonoses, Livestock, Infectious disease reservoirs, Viral hepatitis

## Abstract

**Background:**

Hepatitis E virus (HEV) is a major cause of human hepatitis worldwide. Zoonotic genotypes of the virus have been found in diverse animal species with pigs playing a major role. Putative risk of zoonotic infection from livestock particularly swine in Sub-Saharan Africa including Ghana is poorly understood due to scarcity of available data, especially HEV sequence information.

**Methods:**

Serum samples were collected from cattle, sheep, goats and pigs from Kumasi in the Ashanti region of Ghana. Samples were subjected to nested RT-PCR screening and quantification of HEV RNA-positive samples using real-time RT-PCR and the World Health Organization International Standard for HEV. Testing of all pig samples for antibodies was done by ELISA. Sanger sequencing and genotyping was performed and one representative complete genome was generated to facilitate genome-wide comparison to other available African HEV sequences by phylogenetic analysis.

**Results:**

A total of 420 samples were available from cattle (*n* = 105), goats (*n* = 124), pigs (*n* = 89) and sheep (*n* = 102). HEV Viral RNA was detected only in pig samples (10.1%). The antibody detection rate in pigs was 77.5%, with positive samples from all sampling sites. Average viral load was 1 × 10^5^ (range 1.02 × 10^3^ to 3.17 × 10^5^) International Units per mL of serum with no statistically significant differences between age groups (≤ 6 month, > 6 months) by a T-test comparison of means (t = 1.4272, df = 7, *p* = 0.1966). Sequences obtained in this study form a monophyletic group within HEV genotype 3. Sequences from Cameroon, Ghana, Burkina Faso and Madagascar were found to share a most recent common ancestor; however this was not the case for other African HEV sequences.

**Conclusion:**

HEV genotype 3 is highly endemic in pigs in Ghana and likely poses a zoonotic risk to people exposed to pigs. HEV genotype 3 in Ghana shares a common origin with other virus strains from Sub-Saharan Africa.

## Background

Hepatitis E virus (HEV) is a major cause of hepatitis in humans worldwide. HEV is a single stranded RNA virus with a genome size of approximately 7.2 kb. According to the International Committee on Taxonomy of viruses (ICTV), it is classified into the family *Hepeviridae*. Most of the HEV strains infecting humans belong to the virus species Orthohepevirus A (Genus *Orthohepevirus*), which comprise eight genotypes (gt), of which 1 to 4 and 7 have been found in humans. Genotypes 1 and 2 seem to be restricted to humans while the other three human genotypes are zoonotic and occur in other animals, including pigs (gt3–6), rabbits (gt3) and camelids (gt7) [1–4]. HEV gt1 and gt2 are transmitted faeco-orally and have been responsible for outbreaks in low socioeconomic settings, particularly in Africa [5, 6]. Major risk factors for zoonotic transmission of HEV include contact with infected animals especially pigs and consumption of undercooked animal products [4, 7]. High detection rates of HEV RNA in pigs at slaughter have been demonstrated in different countries with up to 41% in Canada, and up to 44% in the UK [8–11]. This places people like pig handlers and abattoir workers at high risk of infection [12].

Zoonotic subtypes of HEV have also been detected in other major livestock, such as sheep, goats and cattle [13–15]. The role these species play in zoonotic transmission has not been extensively explored and is therefore not known whether these livestock species also serve as natural reservoirs or were accidentally infected by swine derived strains [16].

Waterborne transmission of Genotypes 1 and 2 are believed to be the main cause of HEV infection in endemic regions of Africa, including Ghana [11]. However, extensive knowledge of HEV types and their abundance in livestock species in Ghana and Sub-Saharan African is scarce [17] and as such the putative risk of zoonotic infection from livestock particularly swine in these regions have not been well understood. The purpose of this study was to determine the occurrence and diversity of HEV in major livestock species in a developing country.

## Materials and methods

### Sample collection and RNA extraction

**Fig. 1:**
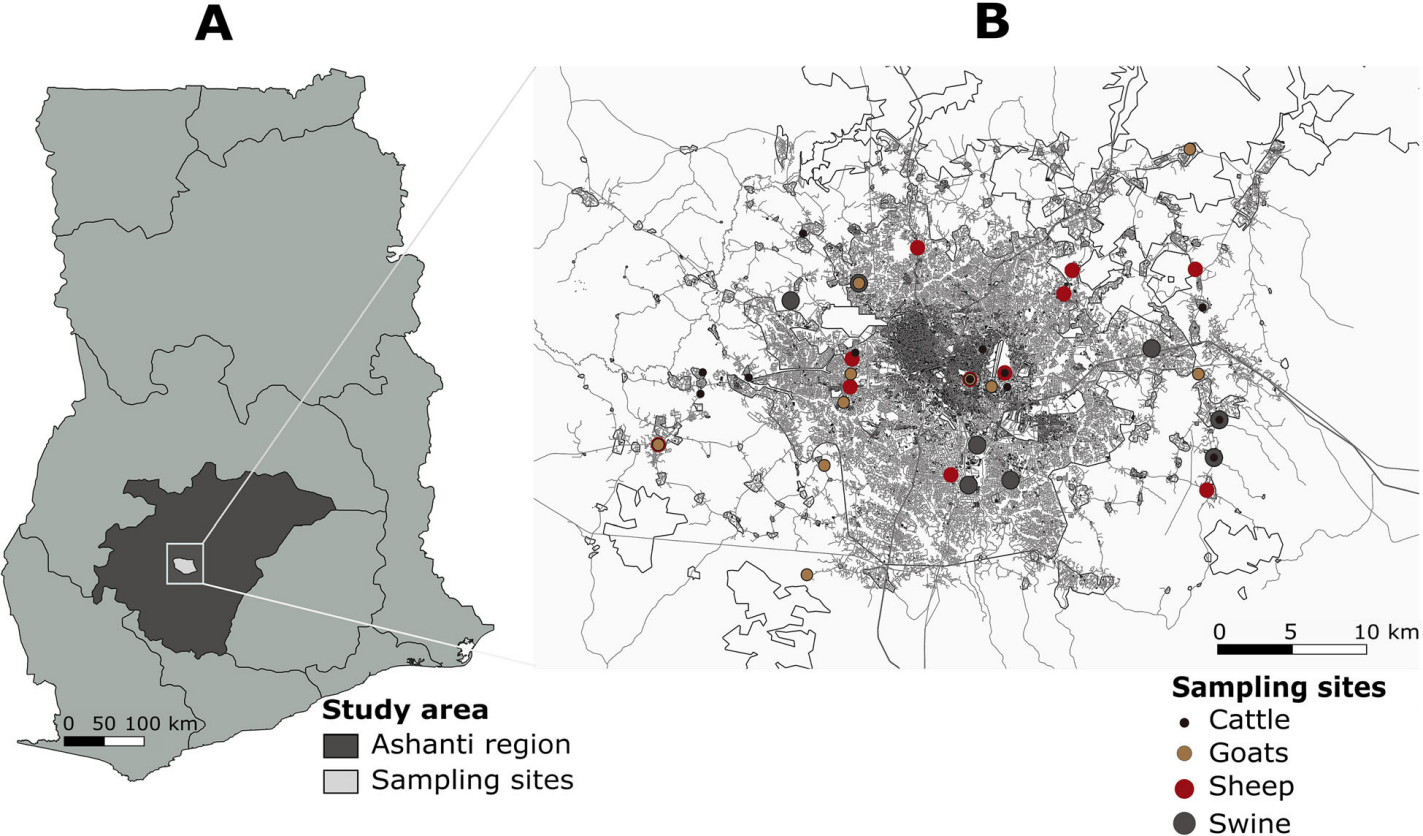
Distribution of samples collected in the study. **a** Map of Ghana. Livestock samples were collected in the Ashanti region around the Kumasi Metropolis (**b**). GPS coordinates from the various sampling sites were plotted using Quantum GIS version 3.6.2 and data freely available from openstreetmap.com. Vector map data on Ghana was obtained from www.diva-gis.org. Coloured dots indicate the sampling locations of the different livestock species

Serum samples from four major domestic livestock namely cattle, sheep, goats and swine were collected from Kumasi in the Ashanti region of Ghana in December, 2011 (Fig. 1) [18].

Blood was collected by venipuncture from swine, goats, sheep, and cattle into clot activation tubes by veterinary officers and centrifuged the same day to obtain serum. Serum was stored at − 20 °C until testing. Viral RNA was extracted from serum in pools of six with a total starting volume of 120 μl using the MagNaPure96 nucleic acid extraction system (Roche Diagnostics Ltd., Risch-Rotkreuz, Switzerland) with the Viral NA Small Volume Kit. Elution volume was 100 μl.

### HEV-RNA detection and complete genome characterization

Detection and sequencing of HEV-RNA was done as previously described [19]. Briefly, we applied a hemi-nested reverse transcriptase (RT)-PCR using broadly reactive primers targeting a 338-nucleotide fragment of the RNA-dependent RNA polymerase (RdRp) gene of HEV with the Superscript III one-step RT-PCR kit (Thermo Fischer, MA, USA). All pools that tested positive by RT-PCR were resolved by testing the component samples individually. Quantification of viral RNA in positive samples was performed by real-time RT PCR as described previously [20, 21]. Standard curve generation for quantification was done using the World Health Organization International Standard for HEV [22].

The complete genome of one representative swine HEV from Ghana was obtained using hemi-nested RT-PCR assays targeting overlapping regions of HEV-3 followed by Sanger sequencing (Microsynth Seqlab, Göttingen, Germany). Oligonucleotides and protocols used for the whole genome sequence generation were published by our group elsewhere [23].

### Serological testing

To investigate past HEV exposure of pigs, all pig sera were tested by a commercial Anti-HEV IgG ELISA (PrioCHECK® HEV Ab porcine ELISA kit; Thermo Fischer, MA, US) according to manufacturer’s instructions. Briefly, buffer-diluted serum samples were incubated for 1 h at 37 °C followed by conjugate incubation for 30 min also at 37 °C. Detection was done with Chromogenic TMB for 30 min at 22 °C.

For antibody testing of HEV-RNA-negative livestock species, a species-independent Anti-HEV ELISA capable of capturing total antibodies (IgG, IgM and IgA) (DRG Instruments GmbH. Marburg, Germany) was used according to manufacturer’s instructions. Positive outcomes were confirmed by serum IgG detection using a more specific recombinant, indirect, immunofluorescence assay (IFA) based on transfected African green monkey kidney cells (Vero B4) expressing a truncated HEV gt 7 capsid. All sera were tested at a dilution of 1:80. Secondary antibody detection for cattle, pigs, sheep and goats was done using Alexa Fluor 488-conjugted goat anti-bovine, goat anti-swine, donkey anti-sheep, and donkey anti-goat antibodies (Dianova GmbH, Hamburg, Germany) which have been shown previously to work for these species [24, 25].

### Data analysis

Descriptive and inferential statistical analysis were performed using R statistical package version 3.6.0 (R Core Team, Vienna, Austria). Multiple sequence alignments were performed as translation alignments using Geneious Prime 2019 (https://www.geneious.com). Recombination analyses were made using RDP4 [26].

Phylogenetic analyses was done by Bayesian inference with Bayesian Evolutionary Analysis by Sampling Trees (BEAST) [27] programme version 1.10.4 using a General time reversible nucleotide substitution model with a Gamma distribution across sites and proportion of invariable sites. A Markov chain Monti Carlo sampling approach with a chain length of 10,000,000 sampled every 1000 steps and the default constant size coalescent population model was used. Final trees were annotated and visualized with TreeAnnotator and FigTree from the same BEAST package.

Subtyping of the full genome sequence was done by phylogenetic comparison with proposed full genome subtypes from other studies.

## Results

### Samples collected

A total of 420 samples were available from cattle (*n* = 105), goats (*n* = 124), swine (*n* = 89) and sheep (*n* = 102). For cattle, goats, and sheep, majority of the individual animals were more than 1 year old. However, for pigs, majority of the sampled animals were below 1 year (Table 1). For all species, more female animals than males were sampled with a combined proportion of 67.4% (Table 1).

**Table 1:** Characteristics of samples collected in the study

	**Livestock Sampled (N, %)**				
	**Cattle (105)**	**Goats (124)**	**Pigs (89**)	**Sheep (102**)	**Total (420)**
**Age categories**					
< 1 year	6 (5.7)	12 (9.7)	83 (93.3)	8 (7.8)	109 (26.0)
> 1 year	99 (94.3)	112 (90.3)	6 (6.7)	94 (92.2)	311 (74.0)
**Sex**					
Male	41 (39.1)	30 (24.2)	39 (43.8)	27 (26.5)	137 (32.6)
Female	64 (60.9)	94 (75.8)	50 (56.2)	75 (73.5)	283 (67.4)

*N* Number

### Testing for HEV RNA and antibodies

RT-PCR testing of all livestock serum samples, resulted in 9 swine serum samples identified to be positive for HEV viral RNA, representing 10.1% of the collected swine samples. Pigs sampled in 3 of the 8 swine sampling sites showed acute HEV infection. No other tested livestock species were found to be positive by the screening RT-PCR. To further estimate the importance of pigs as an HEV reservoir in the sampling region, we further investigated past HEV exposure of pigs to HEV. A total of 69 (77.5%) swine samples from all sampling sites were found to be positive for HEV IgG antibodies by ELISA testing. Out of the 9 HEV-RNA positive samples, 8 were also seropositive; which indicates that the presence of IgG does not rule out the presence of HEV RNA and suggests that the detected antibodies might not provide sterile immunity. Swine ages ranged from 4 months to 36 months with a median age of 6 months. Seroprevalence and RNA detection rate didn’t differ between the age groups of 6 months or younger and older than 6 months (Fisher’s exact test; *p* > 0.9 and *p* > 0.7 respectively, Table 2).

**Table 2:** Determination of associations between test outcomes and age categories of sampled pigs

**Test performed**	**Outcome** **N (%)**	**Age group** **N (%)**		**Fisher’s exact test (*P* value)**
		**≤ 6 months**	**> 6 months**	
**HEV RNA**	Positive (9, 10.1%)	6 (10.7%)	3 (9.0%)	> 0.9
	Negative (80, 89.9%))	50 (89.3%)	30 (90.9%)	
**HEV IgG**	Positive (69, 77.5%)	44 (78,6%)	25 (75.8%)	0.7964
	Negative (20, 22.5%)	12 (21.4%)	8 (24.2%)	

*N* Number

Average viral load as determined by quantitative real time RT-PCR calibrated using the WHO standard for HEV Viral RNA concentrations was 1 × 10^5^ (range 1.02 × 10^3^ to 3.17 × 10^5^) International Units per mL of serum (Table 3), with no statistically significant differences between age groups (T-test comparison of means, *t* = 1.4272, df = 7, *p* = 0.1966, Table 3).

**Table 3:** Serum viral loads of HEV positive pigs and comparison between age categories

**Pig sample**	**Viral Load (IU/mL)**		
GHS 02	3.17 × 10^5^		
GHS 07	8.15 × 10^4^		
GHS 10	5.05 × 10^4^		
GHS 74	9.14 × 10^4^		
GHS 78	1.37 × 10^5^		
GHS 81	2.07 × 10^5^		
GHS 86	1.02 × 10^3^		
GHS 90	8.00 × 10^3^		
GHS 91	1.05 × 10^5^		
**Age group in months**	**Minimum**	**Mean**	**Maximum**
< 6	8.00 × 10^3^	1.27 × 10^5^	3.17 × 10^5^
> 6	1.02 × 10^3^	4.76 × 10^4^	9.14 × 10^4^
All	**1.02 × 10**^**3**^	**1.00 × 10**^**5**^	**3.17 × 10**^**5**^

**Fig. 2:**
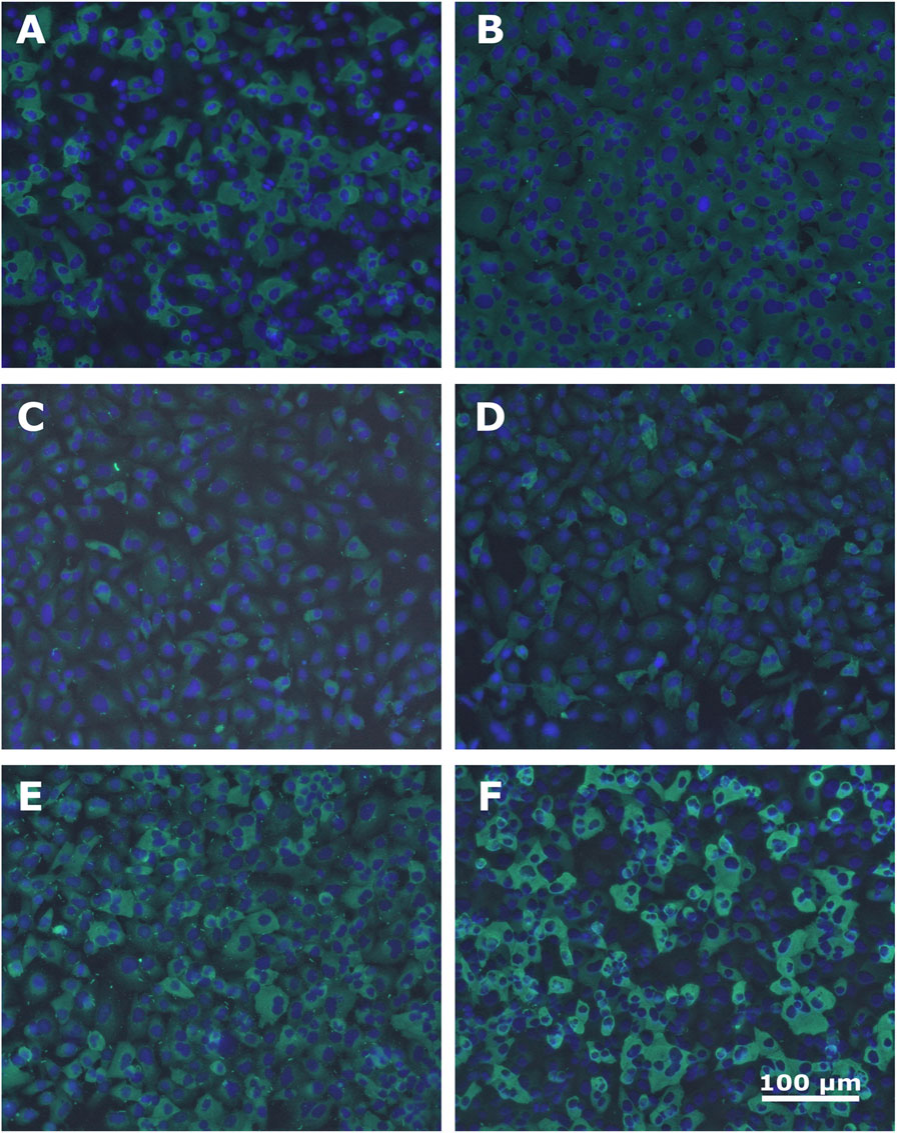
Immunofluorescent detection of HEV IgG antibodies in livestock species. **a** depicts an ELISA positive camel sample showing a positive signal. **b** shows a cattle sample from this study with a negative outcome. **c** and **d** depict IFA-positive goat samples obtained in this study and (**e** and **f**) show two ELISA positive pigs from this study also showing positive IFA signals. Cell nuclei were stained with DAPI and are shown as dark blue and the bright green impressions around the nuclei represent fluorescent antibody-antigen complexes

Among the RNA-negative livestock species, 25 cattle, 13 sheep and 7 goats tested positive for HEV-antibodies using the species-independent ELISA, however only 2 positive outcomes in goats from the same sampling site were confirmed by the immunofluorescence assay (Fig. 2c and d). The performance of the IFA was assessed by the inclusion of a known camel positive sample (Fig. 2a) and two of the ELISA positive swine samples from this study all of which tested positive (Fig. 2e and f). The detection of both camel gt7 and pig gt3 confirms the capability of the IFA to detect antibodies to different genotypes of HEV which may be present in the different livestock species.

### Sequence analyses

**Fig. 3:**
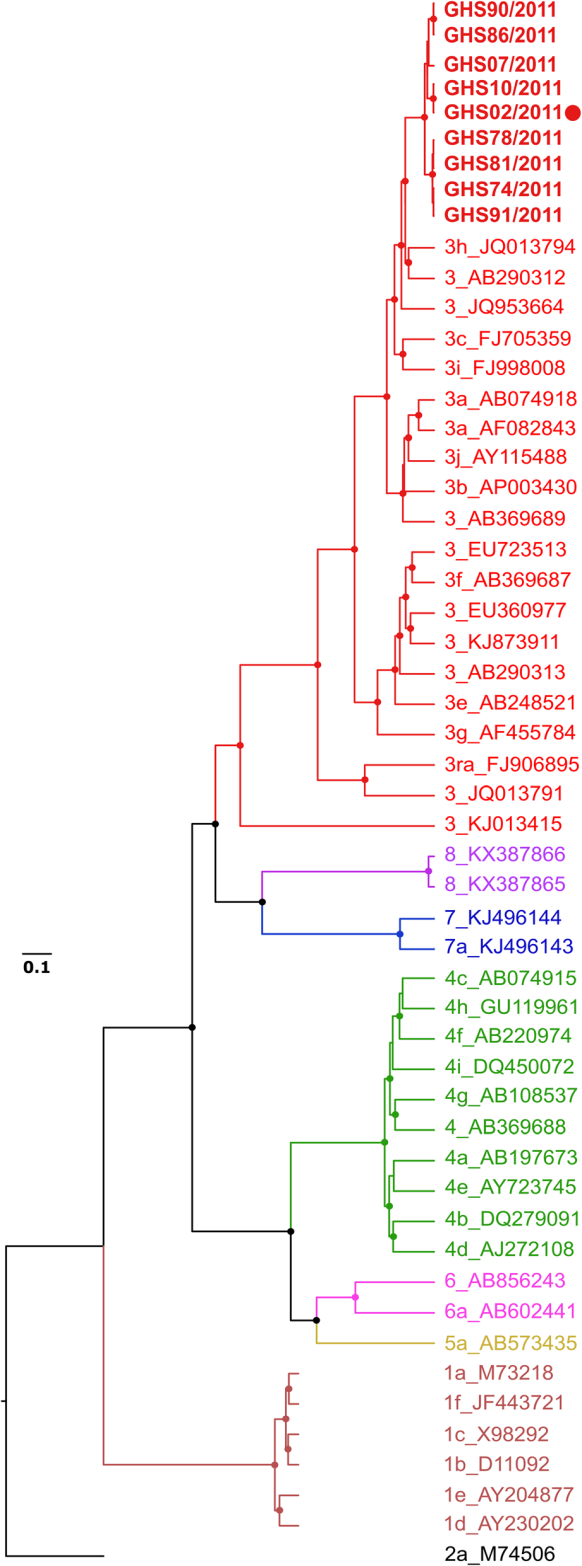
Phylogenetic analysis of sequences obtained in the study in comparison to reference sequences from GenBank. The figure depicts clustering of the various genotypes of HEV with genotype 1 shown in brown, genotype 3 in red, genotype 4 in green, genotype 5 in yellow, genotype 6 shown in purple, genotypes 7 in blue and genotype 8 in violet. Circular nodes represent branching with posterior probabilities greater than 0.95. The tree was rooted with a genotype 2 sequence shown in black. The sequences obtained in this study are identified by sequence specific names in bold type font and clustered with other genotype 3 viruses. The full genome sequence is indicated by a red dot

**Fig. 4:**
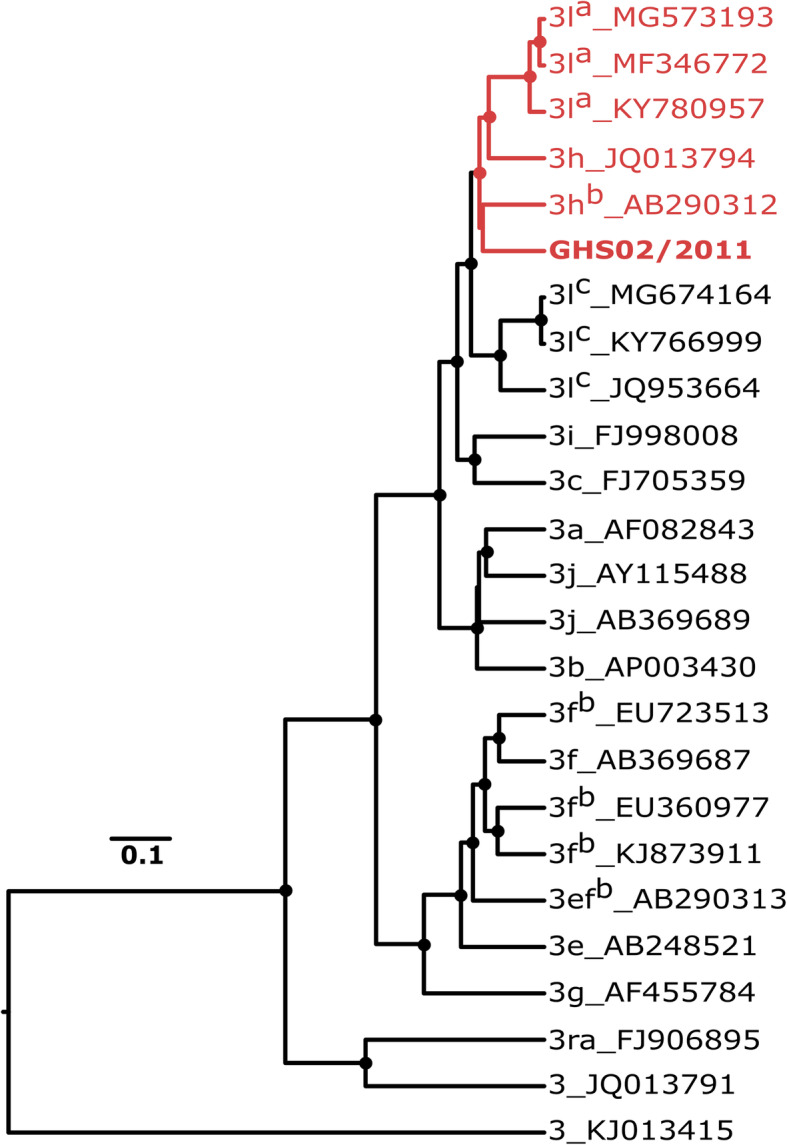
Phylogenetic analysis of the full-length sequence obtained in the study in comparison to proposed full length genotype 3 subtypes. The figure depicts clustering of various genotype 3 subtypes. Circular nodes represent branching with posterior probabilities greater than 0.95. Subtypes indicated are as proposed by Smith et al., [2]. Sequences included by Smith et al., but with unassigned subtypes were assigned subtypes (indicated by superscripts) proposed by Wang et al. (a) [29] Vina-Rodriguez et al., (b) [28] and De Sabato et al., (c) [30]. The sequence obtained in this study was identified by a bold type font and clustered with subtype 3 h and 3l^a^ sequences

For all HEV-RNA positive samples, a 316-nucleotide fragment from the RdRp gene, within Open Reading Frame (ORF) 1 of HEV, was sequenced. A phylogenetic analyses together with reference sequences defined by Smith et al. [2] confirmed all HEV strains from this study to belong to one distinct monophyletic group within HEV gt3 (Fig. 3). For detailed genomic characterization and to provide an avenue for comparison with other studies on African HEV sequences targeting different regions of the genome, we selected one sample with the highest RNA concentration for full-length genome sequencing. The obtained HEV sequence showed typical genome organization for Orthohepevirus A strains, including the presence of short 3′ and 5′ untranslated regions at the genome termini and presence of three predicted open reading frames (ORF1, ORF2, and ORF3). There was no evidence hinting to recombination of the Ghanaian pig HEV strain with other Orthohepevirus species or the known Orthohepevirus A subtypes. A phylogenetic analysis using HEV gt3 subtypes proposed by smith et al., [2] Vina-Rodriguez et al., [28] Wang et al., [29] and De Sabato et al., [30] showed the full genome sequence from this study appeared to belong to the subtype 3 h (Fig. 4). All sequences were submitted to GenBank and assigned accession numbers MN714358 to MN714366.

The Ghanaian pig HEV shares an 87.5% nucleotide identity averaged over the full genome with the most closely related sequence strain from Mongolia (GenBank Acc No: AB290312) followed by a sequence from France (86.7%, GenBank Acc No: JQ013794). Partial sequences obtained in the study had sequence identities ranging between 87.7 to 89.9% and with the exception of GHS07 which was also found to be most closely related to the sequence from Mongolia, all other partial sequences were found to be most closely related to a sequence from Germany (Acc No: FJ998008) (Table 4). A partial section of the full genome in the same region as the other partial sequences was also found to be most closely related to the sequence from Germany. This hints to the likelihood of all other partial sequences being more closely related to the sequence from Mongolia at the full genome level.

**Table 4:** Sequence identities of partial sequences obtained in the study

Sequence ID	Sequence Identity (%)	Most closely related reference sequence (Accession number)
GHS07	88.6	AB290312
GHS10	88.3	FJ998008
GHS74	89.9	FJ998008
GHS78	89.6	FJ998008
GHS81	89.2	FJ998008
GHS86	87.7	FJ998008
GHS90	87.7	FJ998008
GHS91	89.6	FJ998008

To test if all African HEV gt3 sequences form a single monophyletic group and might stem from a common ancestor, phylogenetic analyses of the RdRp region within the ORF1 and the capsid region within ORF2 were done by adding all publicly available sequences from Africa available in GenBank (as of 15th November 2019) to our analyses. Although sequences from Cameroon, Ghana, Burkina Faso and Madagascar did share a most common recent ancestor in capsid or RdRp based phylogeny (Figure S1 to S2) other African HEV sequences from Nigeria, Democratic Republic of the Congo, Uganda, and São Tomé and Príncipe did not cluster in the same monophyletic group and intermixed with other non-African HEV strains (Figure S1 to S2).

## Discussion

Hepatitis E virus is known to be circulating in Ghana with previous studies focused on humans reporting seroprevalences in people exposed to livestock and immunocompromised individuals [31, 32]. In Ghana, HEV infections in pregnant women has been found to be a serious cause of perinatal morbidity and mortality [33]. Exposure to HEV among pigs and humans appears to significantly vary between regions with one study estimating around 4.6% among blood donors in the Ashanti region [34] but up to 58% among community members and 88% in pigs in the Upper East region as against the 77.5% in pigs observed in this study [35]. Among the viral causes of hepatitis in Ghana, HEV appears to be second only to Hepatitis B [36]. Data on prevalence and knowledge of circulating genotypes of HEV in the livestock population is however widely scarce due to lack of studies reporting sequence information [37].

Beyond Ghana, HEV has been found in domestic and wild animals from different parts of the world [38]. Without totally excluding domestic livestock like sheep, goats, cows and equids, swine have been found to be the main source of human infections worldwide [12–15, 37, 39, 40]. In line with these findings, cattle, sheep, and goats were not found to harbour any active HEV infections and also exhibited a low level of previous exposure in our study. The fact that swine samples from all sampling sites were found to be positive for HEV IgG antibodies suggests a high infection rate in pigs independent of the farm of origin and are consistent with reports of HEV infection in pigs in Europe, Asia, and the Americas [11], hinting to a similar epidemiology of HEV gt3 in pigs worldwide.

Detection of HEV RNA among swine can be considered a measure of the risk to people in contact with swine or swine products [41]. The detection rate however varies based on organs or tissues tested with the detection rate of the virus in blood generally lower than that from other organs like liver or in faeces and caecal contents [42–44].

Detection rate in blood in this study was higher than in other studies conducted in Asia, Europe and South America [42, 45–48] possibly as a consequence of differences in husbandry practices [49] between sub-Saharan Africa and other parts of the world. However, the average HEV viral load determined in this study was within the range of another study conducted in the United Kingdom which found a range from as low as below 100 IU/mL to as high as 10^6^ IU/mL in plasma at slaughter [42]. Studies comparing infective doses of the different HEV strains are lacking [6] however as a general indicator for risk of zoonotic transmission, the viral load has been found to influence the potential of getting infected [12].

Age of detection of viremia in swine has also been found to range from 2 to 6 months in previous studies from different parts of the world [10, 50, 51] but was slightly higher in this study. As the age of slaughter is mostly below 1 year in many parts of the world including Ghana, the risk of infection therefore also appears comparable worldwide [10, 52–54]. Adjusting slaughter age to periods after this may help reduce the risk of human infection, as also suggested in another study from France that found a lower HEV RNA prevalence in pigs older than 6 months as compared to those that were 3–4 months old [55].

Prevalence of seroconverted pigs reported varies widely and are difficult to compare due to variations in sensitivities of testing methods. However the antibody detection rate reported in this study was comparable to those from other studies worldwide [11].

In developed countries, eating raw or undercooked pork is a major cause of zoonotic transmission of HEV [56]. In Ghana, consumption of raw pork is not common due to cultural consumption norms. The main risk of zoonotic transmission is therefore more likely to be due to exposure to pigs and pig products in occupationally exposed people like slaughterhouse workers. This risk of zoonotic transmission of HEV gt3 from pigs to humans is underlined by the remarkable RNA detection rate of 10% in serum and the high virus concentration in these samples.

The fact that sequences from this study were found to be most closely related to genotype 3 sequences from other West African countries (Cameroon and Burkina Faso) might hint to a common origin of some African swine genotype 3 HEV viruses. However, the clustering of African gt3 HEV viruses in different monophyletic groups challenges this hypothesis. This inconsistency can however be explained by the fact that trade of live pigs and their HEV strains is common and pigs were regularly imported into Africa from Europe and Asia in previous times [57].

## Conclusions

In summary, HEV genotype 3 appears to be the main genotype circulating in pigs from Ghana with similar infection dynamics to those observed in other parts of the world. The geographic origin of these viruses could be from within sub-Saharan Africa but more studies reporting sequences are needed to assess this. Viral loads in slaughter age pigs in this study point to a high risk of infection among slaughterhouse workers or people with close contact to pigs in Ghana.

## Supplementary information

**Additional file 1: Figure S1.** Cladogram based on partial and full-length sequences from the HEV gt3 capsid region. **Figure S2.** Cladogram based on partial and full-length sequences from the HEV gt3 RdRp region.

## Data Availability

Data generated or analysed during this study are included in this published article and its supplementary information files. Sequences are available via NCBI GenBank. Further supporting data if required are available from the corresponding author on reasonable request.

## Abbreviations

BEAST: Bayesian Evolutionary Analysis by Sampling Trees
ELISA: Enzyme-Linked Immunosorbent Assay
GIS: Geographic Information System
GPS: Global Positioning System
GT3: Genotype 3
HEV: Hepatitis E Virus
ICTV: International Committee on Taxonomy of Viruses
IFA: Immunofluorescence assay
IgA: Immunoglobulin A
IgG: Immunoglobulin G
IgM: Immunoglobulin M
IU: International Units
MA: Massachusetts
mL: Millilitre
NA: Nucleic acid
ORF: Open Reading Frame
PCR: Polymerase Chain Reaction
RDP4: Recombination Detection Program 4
RdRp: RNA-dependent RNA polymerase
RNA: Ribonucleic Acid
RT: Reverse Transcriptase
TMB: 3,3′,5,5′-Tetramethylbenzidine
UK: United Kingdom
USA: United States of America
WHO: World Health Organization
