# Supplementary material for: Detection and genomic characterization of hepatitis E virus genotype 3 from pigs in Ghana, Africa
Source: One Health Outlook. 2020 Jul 20;2:10. doi: 10.1186/s42522-020-00018-3 (PMC7993477; doi:10.1186/s42522-020-00018-3)
Supplement: Supplementary file 1 — Additional file 1: Figure S1. Cladogram based on partial and full-length sequences from the HEV gt3 capsid region. Figure S2. Cladogram based on partial and full-length sequences from the HEV gt3 RdRp region. [file 42522_2020_18_MOESM1_ESM.docx]

**Appendix**


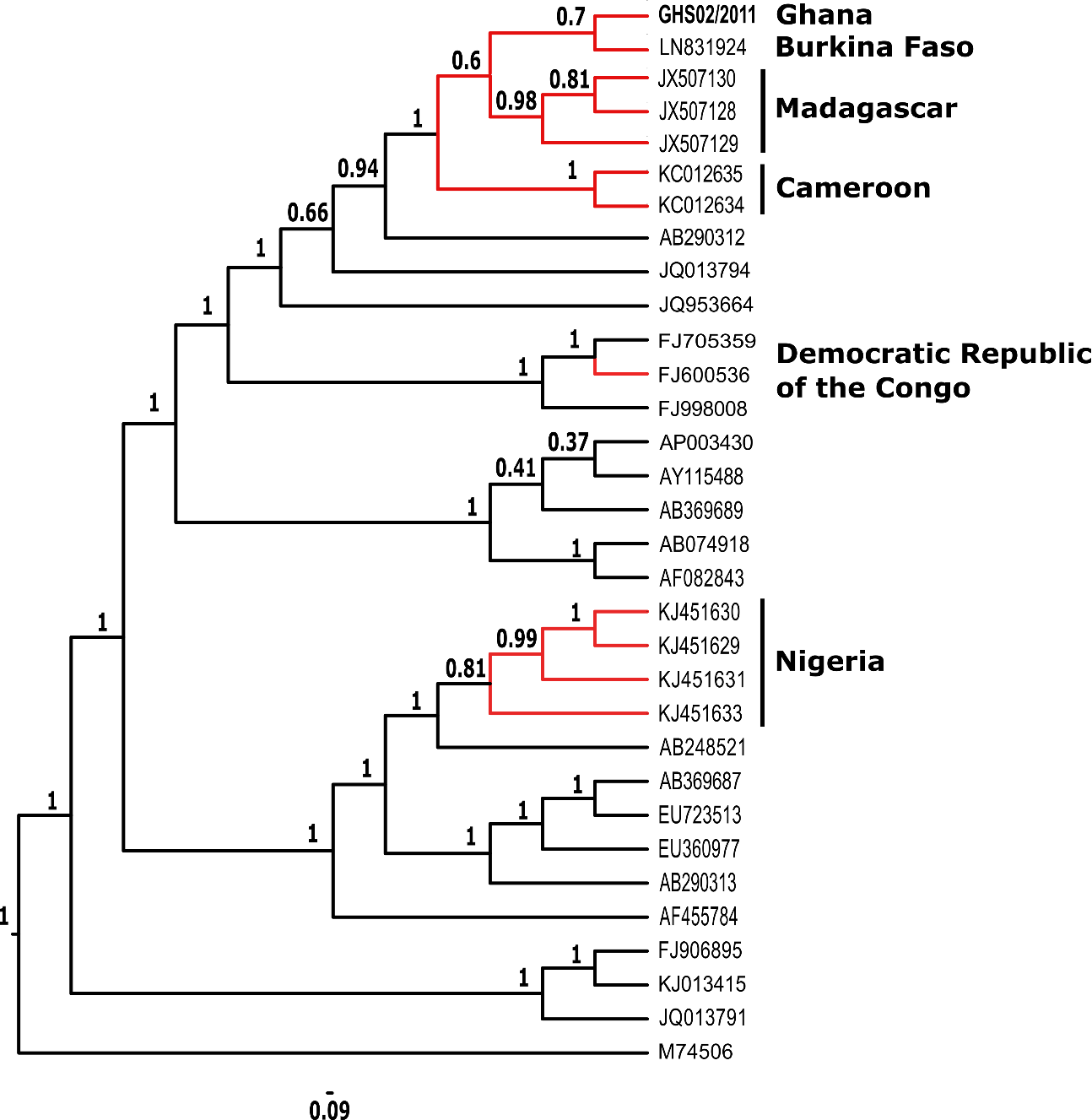


**Figure S 1. Cladogram based on partial and full-length sequences from the HEV gt3 capsid region**

African sequences are depicted with red branches and the country of origin indicated next to sequence accession numbers. Trees were rooted with a genotype 2 sequence and the full-length sequence obtained in this study is highlighted by a bold type font. The tree depicts clustering of African sequences in different monophyletic groups.


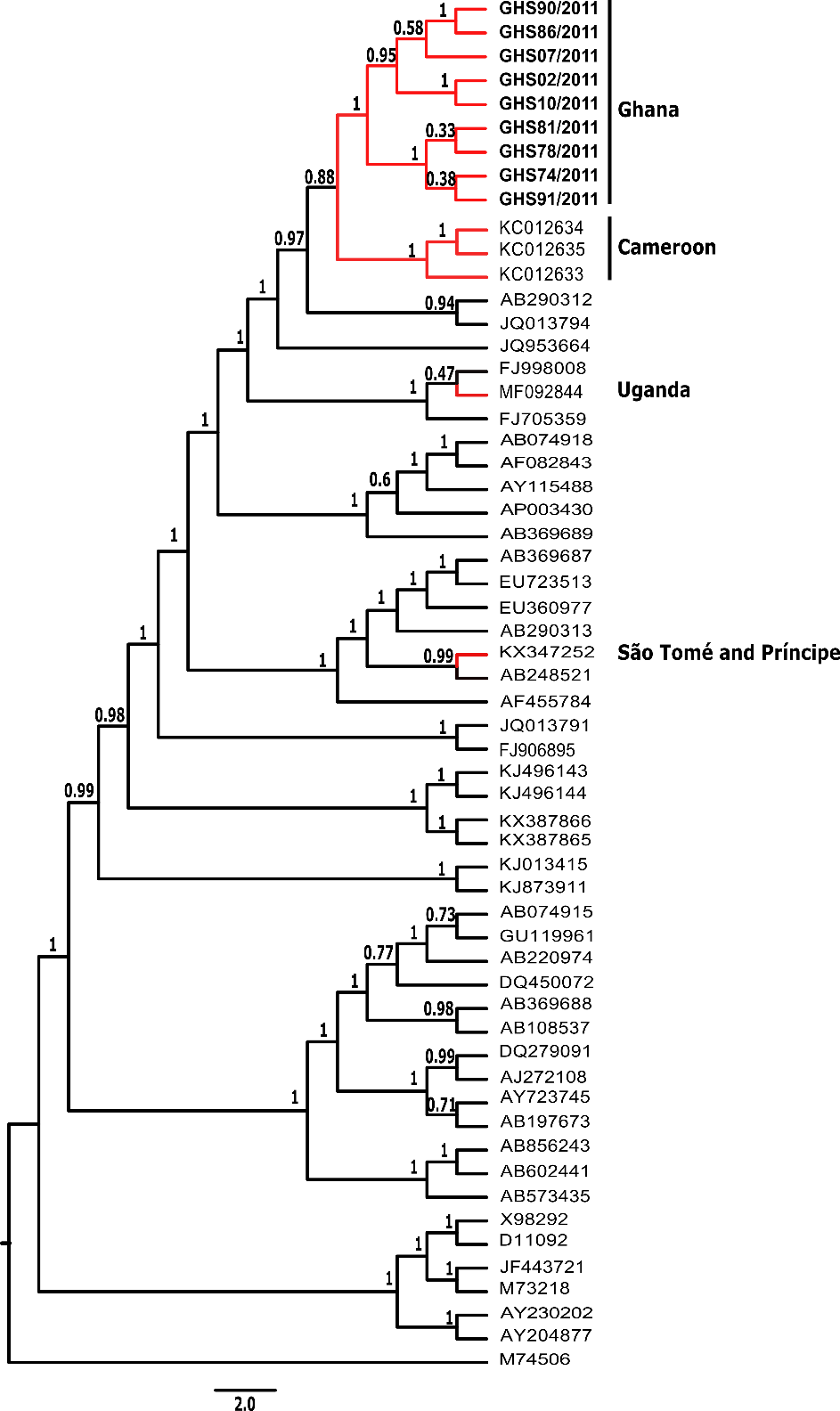


**Figure S2. Cladogram based on partial and full-length sequences from the HEV gt3 RdRp region**

African sequences are depicted with red branches and the country of origin indicated next to sequence accession numbers. Trees were rooted with a genotype 2 sequence and the sequences obtained in this study are highlighted by a bold type font. The tree depicts clustering of African sequences in different monophyletic groups.
